# Prevalence and predisposing factors of depressive symptoms in continuous ambulatory peritoneal dialysis patients: a cross-sectional single center study

**DOI:** 10.1186/s12882-023-03166-6

**Published:** 2023-04-21

**Authors:** Yu Chen, Peng Li, Lei Zhang, Yanfei Zhang, Luyi Xie, Jianying Niu

**Affiliations:** Department of Nephrology, Shanghai Fifth People’s Hospital, Fudan University, 801 Heqing Rd, Shanghai, 200240 China

**Keywords:** Depressive symptoms, Peritoneal dialysis, Serum hemoglobin

## Abstract

**Background:**

The aim of this study was to identify the prevalence of the depressive symptoms and the factors associated with the depressive symptoms in peritoneal dialysis patients.

**Methods:**

A cross-sectional study was carried out to evaluate the prevalence and associated factors of depression in 132 continuous ambulatory peritoneal dialysis patients. Depression was evaluated using Zung Self-Rating Depression Scale. Sociodemographic and clinical characteristic were also investigated. Univariate analysis and multivariate logistic regression analysis were performed to select factors associated with depressive symptoms.

**Results:**

Their median age was 57.5 years, and 58.3% were male. The rate of depressive symptoms in peritoneal dialysis patients was 78.0%. The rate of moderate/severe depressive symptoms was 64.4%. Multivariable logistic regression analysis showed that lower serum hemoglobin was significantly associated with increased risks of depression (OR = 0.989, 95CI%=0.979–0.998, *p* = 0.023).

**Conclusion:**

Depression was highly prevalent in the peritoneal dialysis patients. Serum hemoglobin was independent risk factor for depressive symptoms in peritoneal dialysis patients.

## Background

Approximately 11.0–98.5% of peritoneal dialysis patients have been afflicted by depression [1–5], which are significantly higher than that of the general population. Prevalence of depression in peritoneal patients was also significantly higher than that in the chronic kidney disease patients without dialysis in China [2, 6, 7].

The occurrence of depressive disorder in end-stage renal disease patients was associated with the assessment tool for screening depression and many sociodemographic and clinical characteristics, such as sex, age, occupation, malnutrition, comorbidity, and time since diagnosis [4, 8–11].

Depression in dialysis patients is associated with higher risk of mortality, greater use of healthcare costs, and hospitalization, and poorer treatment adherence [12–15]. In addition, the depression in peritoneal dialysis patients has been associated with higher incidence of peritonitis [16]. Indeed, the 2020 International Society for Peritoneal Dialysis practice recommendations have already emphasized the need to screen mood disorder (including depression) in continuous ambulatory peritoneal dialysis (CAPD) patients [17].

However, the factors associated with depression in CAPD patients remain poorly understood. The purpose of our study was to investigate the prevalence of depressive patients and determine the risk factors linked with depressive symptoms by comparing sociodemographic and clinical data among those patients to clarify the relationship between peritoneal dialysis and depressive symptoms.

## Methods

### Study design and population

**Fig. 1:**
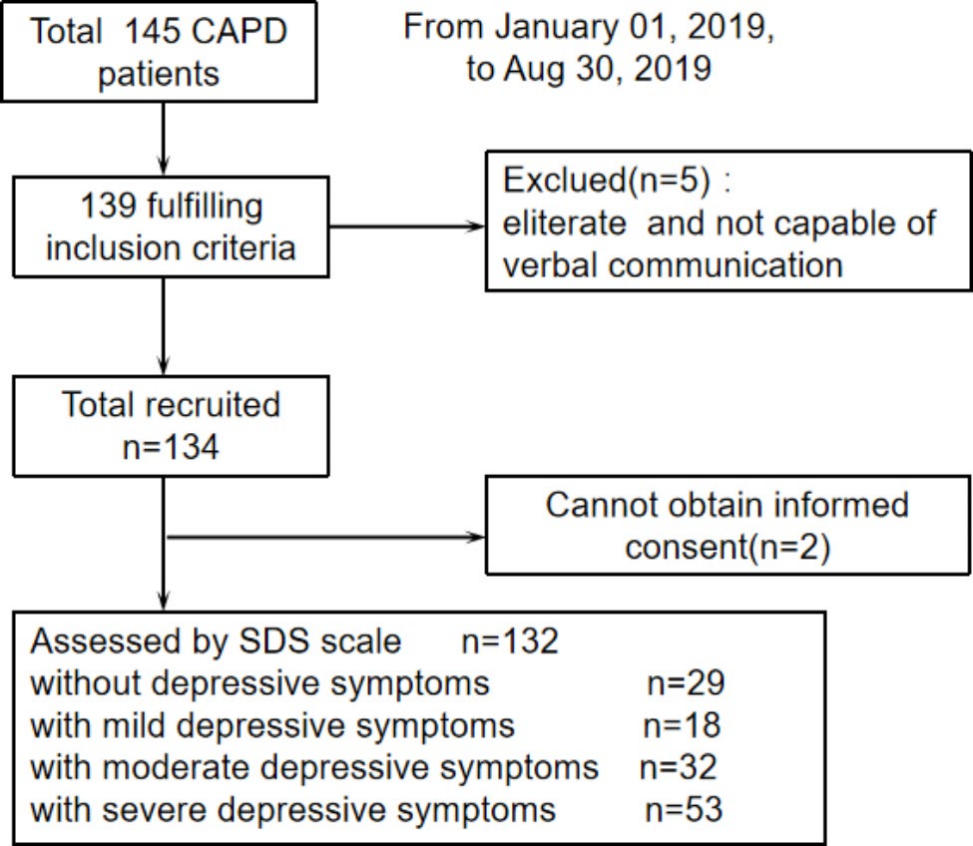
Patient flow chart for the study enrollment for continuous ambulatory peritoneal dialysis patients

The study was a cross-sectional, observational, and single-center study. The study population consists of one hundred and thirty-two peritoneal dialysis patients aged over 18 years with end-stage renal disease receiving continuous ambulatory peritoneal dialysis for at least 3 months. Study subjects were recruited between January 01, 2019, and Aug 30, 2019. Patients who were illiterate and not capable of verbal communication were excluded. Eligible patients were provided details about the assessment procedure (as shown in Fig. 1).

**Table 1:** Characteristics of continuous ambulatory peritoneal dialysis patients with or without depressive symptoms

Characteristics of patients	Non-depressive symptoms group	Depressive symptoms group	*p* -value
N	29	103	/
Men, n(%)	20(69.0)	57(55.3)	0.189
Age, years	55.0(48.0-60.5)	59.0(48.0–69.0)	0.108
Married	27(93.1)	91(88.3)	0.464
Employed	7(24.1)	12(11.7)	0.092
Current Smoking	3(10.3)	9(8.7)	0.791
Current Alcohol Drinker	2(6.9)	5(4.9)	0.666
CAPD Vintage, months	36.0(15.5–63.5)	26.0(9.0–55.0)	0.095
Hypertension, n(%)	28(96.6)	99(96.1)	0.914
Diabetes Mellitus, n(%)	9(31.0)	51(49.5)	0.077
History of CVD, n(%)	5(17.2)	29(28.2)	0.237
BMI, Kg/m^2^	22.15(20.76–25.25)	22.72(20.70-24.44)	0.727
Systolic blood pressure, mmHg	140(120–160)	140(130–152)	0.923
Diastolic blood pressure, mmHg	82(79–90)	80(70–90)	0.150
Kt/V	1.47(1.26–1.77)	1.73(1.34–2.10)	0.072
Laboratory Values			
Hemoglobin, g/L	110(86–147)	105(90–120)	0.432
Albumin, g/dL	32.8(29.1–38.0)	34.0(30.9–38.0)	0.833
Blood urea nitrogen, mmol/L	21.1(18.4–25.4)	19.9(14.4–24.2)	0.087
Creatinine, µmol/L	995.0(848.5–1194.0)	934.0(707.0-1128.0)	0.151
Total Cholesterol, mg/dL	4.0(3.1–4.9)	3.88(3.13–4.75)	0.836
Triglycerides, mg/dL	1.05(0.78–2.05)	1.36(0.86–2.78)	0.245
HDL-C, mg/dL	0.98(0.87–1.12)	1.08(0.76–1.30)	0.854
LDL-C, mg/dL	2.49(1.81–3.14)	2.03(1.76–2.88)	0.142
C reactive protein, mg/dL	2.97(1.16–5.22)	2.37(0.71–6.48)	0.625
Calcium, mmol/L	2.11(1.96–2.25)	2.18(2.05–2.30)	0.303
Phosphorus, mmol/L	1.85(1.67–2.35)	1.67(1.37–2.12)	0.105
Potassium, mmol/L	4.4(3.4–4.8)	4.1(3.4–4.85)	0.831
Total Bilirubin, µmol/L	6.3(4.1–8.5)	6.2(4.8–8.8)	0.466
Alanine Amino-transferase, U/L	12.9(9.2–19.1)	11.0(7.2–15.9)	0.256
Aspartate Amino-transferase, U/L	14.0(11.5–17.0)	14.3(10.5–19.6)	0.945
Parathyroid hormone, pg/ml	29.7(18.4–46.7)	29.2(13.5–42.5)	0.468
Brainnatriuretic peptide, pg/ml	16,000(3990–35,000)	8440(1980–19,200)	0.883
Medication, n(%)			
CCB	26(89.7)	89(86.4)	0.645
ARB or ACEi	20(69.0)	55(53.4)	0.135
β-receptor Blocker	12(41.4)	57(55.3)	0.184
Aspirin	5(17.2)	21(20.4)	0.707
Weekly dose of Erythropoietin, U	10,000(6000–10,000)	10,000(10,000–10,000)	0.464
Statins	14(48.3)	49(47.6)	0.868

Values expressed as mean ± standard deviation, percentage or median (interquartile range)

Abbreviations: *CAPD* continuous ambulatory peritoneal dialysis, *CVD* cardiovascular disease, *BMI* body mass index, *HDL-C* high-density lipoprotein- cholesterol, *LDL-C* low-density lipoprotein- cholesterol, *CCB* calcium-channel blocker, *ARB* angiotensin II type1 receptor blocker, *ACEi* angiotensin-converting enzyme inhibitor

### Data collection

Demographic and clinical data were obtained through interviews, medical records, and actual measurement, including age, sex, body mass index, work status, marital status, hypertension history, diabetes history, cardiovascular disease history, smoking history, and alcohol drinking history, blood pressure, pre-existing medical conditions and so on. Fasting venous blood samples were collected and sent to our laboratory for measurement of alanine aminotransferase, aspartate aminotransferase, blood total bilirubin, albumins, blood urea nitrogen, creatinine, triglycerides, total cholesterol, high-density lipoprotein- cholesterol, low-density lipoprotein- cholesterol, hemoglobin, brain natriuretic peptide and so on.

**Table 2:** Univariable logistic regression analysis of potential risk factors for depression in continuous ambulatory peritoneal dialysis patients

Parameters	B	OR (95% CI)	*p*-value
Men, n(%)	0.584	1.793(0.746–4.312)	0.192
Age, years	0.029	1.029(0.997–1.063)	0.076
Married	-1.306	3.692(0.460-29.659)	0.219
Employed	-0.881	0.414(0.146–1.175)	0.098
Current Smoking	-0.187	0.830(0.209–3.288)	0.791
Current Alcohol Drinker	-0.373	0.689(0.127–3.749)	0.666
CAPD Vintage, months	-0.008	0.992 (0.980–1.004)	0.172
Diabetes Mellitus	-0.779	0.459(0.191–1.102)	0.081
Hypertension	0.123	1.131(1.122–10.533)	0.914
History of CVD	0.632	1.881(0.655–5.402)	0.240
BMI, Kg/m^2^	-0.065	0.937(0.849–1.034)	0.194
Systolic blood pressure, mmHg	-0.004	0.996(0.978–1.104)	0.644
Diastolic blood pressure, mmHg	-0.017	0.983(0.951–1.015)	0.298
Kt/V	0.639	1.895(0.835–4.301)	0.126
Laboratory values			
Hemoglobin, g/L	-0.011	0.989(0.982–0.997)	0.006*
Albumin, g/dL	-0.010	0.990(0.912–1.074)	0.804
Blood urea nitrogen, mmol/L	-0.040	0.961(0.903–1.022)	0.201
Creatinine, µmol/L	-0.001	0.999(0.998-1.000)	0.106
Total cholesterol, mg/dL	-0.051	0.950(0.685–1.318)	0.759
Triglycerides, mg/dL	0.088	1.092(0.885–1.348)	0.411
HDL-C, mg/dL	0.039	1.039(0.310–3.486)	0.950
LDL-C, mg/dL	0.373	0.689(0.434–1.092)	0.113
C reactive protein, mg/dL	0.020	1.020(0.967–1.077)	0.464
Calcium, mmol/L	0.217	1.243(0.390–3.964)	0.714
Phosphorus, mmol/L	-0.390	0.677(0.346–1.327)	0.256
Potassium, mmol/L	-0.096	0.908(0.544–1.516)	0.712
Total bilirubin, µmol/L	0.019	1.019(0.891–1.166)	0.782
Alanine amino-transferase, U/L	-0.032	0.968(0.907–1.034)	0.341
Aspartate amino-transferase, U/L	0.009	1.009(0.939–1.083)	0.814
Parathyroid hormone, pg/ml	-0.002	0.998(0.989–1.007)	0.662
Brainnatriuretic peptide, pg/ml	-0.010	0.990(0.960–1.022)	0.547
Medication, n(%)			
CCB	0.310	1.363(0.364–5.110)	0.646
ARB or ACEi	0.723	2.060(0.769–5.519)	0.151
β-receptor blocker	-0.563	0.570(0.247–1.313)	0.186
Weekly dose of Erythropoietin, 1000U	0.007	1.007(0.907–1.118)	0.892
Aspirin	-0.206	0.813(0.277–2.386)	0.707
Statins	0.009	1.010(0.442–2.305)	0.982

*Statistically significant at *p*<0.05

Abbreviations: *OR* Odds ratio, *CI* confidence interval, *CAPD* continuous ambulatory peritoneal dialysis, *CVD* cardiovascular disease, *BMI* body mass index, *HDL-C* high-density lipoprotein- cholesterol, *LDL-C* low-density lipoprotein- cholesterol, *CCB* calcium-channel blocker, *ARB* angiotensin II type1 receptor blocker, *ACEi* angiotensin-converting enzyme inhibitor

### Assessment of depression symptoms

The Zung Self-Rating Depression Scale (SDS) was used to screen depressive symptoms [13–15]. SDS is a self-reported clinical scale and contains 20 items. Every item can be scored from 1 (where depressive symptoms are very seldom) to 4 (where depressive symptoms are most of the time). The total score was defined as the sum of the total numbers obtained in the 20 items. And the standardized score is equal to 1.25 times the total score and is 100 points scale. Depressive symptoms were defined as a standardized score of 53 or higher; 53–62 indicates mild depressive symptoms, 63–72 indicates moderate depressive symptoms, and > 72 indicates severe depressive symptoms. The patients were categorized into two subgroups based on the results of SDS: non-depressive symptoms group (score ≤ 52), depressive symptoms group (score ≥ 53).

### Statistical analyzes

Data are expressed as means ± standard deviation for continuous parametric data, medians and interquartile ranges for continuous nonparametric data, and frequencies for categorical data. Potential differences among the two groups were assessed with t-test for normally distributed data, Mann-Whitney U for nonnormally distributed data. Univariable and multivariable logistic regression analysis was used to determine the factors associated with depressive symptoms. The results were presented as the odds ratio (OR) and corresponding 95% confidence interval (CI). All statistical analyses were performed using IBM SPSS software (SPSS Inc., Chicago, USA). A two-tailed *p* < 0.05 indicated statistical significance.

**Table 3:** Multivariable logistic regression analysis of potential risk factors for depression in continuous ambulatory peritoneal dialysis patients

Parameters	B	Adjust OR (95% CI)	*p*-value
Age, years	0.020	1.020(0.976–1.067)	0.375
Employed	0.235	1.265(0.223–7.173)	0.791
Diabetes mellitus	0.953	2.595(0.841–8.006)	0.097
Hemoglobin, g/L	-0.011	0.989(0.979–0.998)	0.023*
Creatinine, µmol/L	-0.001	0.999(0.997-1.000)	0.120
Low-density lipoprotein- cholesterol, mg/dL	-0.341	0.711(0.419–1.206)	0.206

*Statistically significant at *p*<0.05

Abbreviations: *OR* Odds ratio, *CI* confidence interval

## Results

### Patients’ sociodemographic and clinical characteristics

Of 145 CAPD patients at baseline, thirteen patients who did not meet the eligibility criteria were excluded. One hundred and thirty-two patients completed the SDS. The median age was 57.5 years (interquartile ranging from 48 to 68 years). Seventy-seven (58.3%) were male. And most of the participants (89.4%) were married. Unfortunately, the majority of them (85.6%) were unemployed. Among these CAPD patients, median dialysis vintage was 27.5 months. The most common comorbidity (96.2%) was hypertension.

The sociodemographic and clinical characteristics of CAPD patients in the non-depressive symptoms and depressive symptoms groups are shown in Table 1. There were no significant differences in the sociodemographic and clinical parameters between the two groups (*p*>0.05). There were no significant differences in the hemoglobin among no depression group, mild depression group, moderate depression group and severe depression group (*p* = 0.207).

### Prevalence and predictors of depression among CAPD patients

Further analysis showed that 78.0% of CAPD patients had depression. Among the patients with depressive symptoms, 17.5% had mild depression, 31.1% had moderate depression and 51.4% had severe depression.

In univariable logistic analysis, serum hemoglobin was the only variable which had significant association with depression (OR = 0.989, 95CI%=0.982–0.997, *p* = 0.006) (Table 2).

Table 2 shows that diabetes mellitus history (OR = 0.459, *p* = 0.081), patients age (OR = 1.029, *p* = 0.076), employed status (OR = 0.414, *p* = 0.098), hemoglobin (OR = 0.989, *p* = 0.006), creatinine (OR = 0.999, *p* = 0.106) and low-density lipoprotein- cholesterol (OR = 0.689, *p* = 0.113) are the variables with *p* < 0.120 and will be included in the multivariate analysis.

In multivariate logistic regression analysis, serum hemoglobin level was still an independent risk factor for depressive symptoms (OR = 0.989, 95CI%=0.979–0.998, *p* = 0.023, as shown in Table 3). Serum hemoglobin remained significant after adjustment for potentially sociodemographic and clinical confounders in multivariable analysis.

**Table 4:** Summary of prevalence for depressive symptoms in continuous ambulatory peritoneal dialysis patients

Prevalence	Mean ± SD	Men (%)	Sample size	The tools used for diagnosis depression	Criteria for defining depression	Country of study	Reference
62.5%	53.8 ± 14.9	25(52.1%)	48	self-report questionnaire	BDI	Turkey	1
34.0%	49.5 ± 15.7	107(56.0%)	191	self-report questionnaire	BDI-II	China	2
59.2%	/	/	27	clinical interview	HADS	Australia	3
11.0%	53.4 ± 11.2	56 (52.0%)	108	self-report questionnaire	PHQ-9	Thailand	4
98.5%	45.8 ± 15.5	62(46.6%)	133	self-report questionnaire	the Zung SDS	Saudi Arabia	5

Abbreviations: *SD* Standard deviations, *BDI* The Beck Depression Inventory, *BDI-II* The Beck Depression Inventory–II, *HADS* the Hospital Anxiety and Depression Scale, *PHQ-9* The Patient Health Questionnaire, *SDS* Self-Rating Depression

## Discussions

The present study showed the prevalence of depressive symptoms in peritoneal dialysis patients was 78.0%. Furthermore, 64.4% of the peritoneal patients had moderate/severe depressive symptoms i.e. SDS score ≥ 63. This study demonstrated the occurrence of depression was independently associated with serum hemoglobin in CAPD patients.

Recent study suggested that the prevalence of depressive disorder in chronic kidney disease patients was higher using self-rating questionnaire than that using interview-based assessment [8]. The prevalence of depression in the CAPD patients in Turkey was 62.5% evaluated by the Beck Depression Inventory [1]. In Saudi Arabia, the prevalence of depression was 98.5% among PD patients, using the Zung Self-Rating Depression Scale(SDS) [5]. The summary prevalence of depression in continuous ambulatory peritoneal dialysis patients was shown in Table 4. The prevalence of depression in present study was also very high. Firstly, the occurrence of depressive disorder in end-stage kidney disease patients was associated with the tools used for diagnosis and age, occupation, malnutrition, comorbidity and so on [4, 8–11]. The median age in this study was 57.5 years and most of them were elder people. And up to 99.6% patients had coexisting chronic illness, including diabetes mellitus, cardiovascular disease, hypertension. These sociodemographic and clinical characteristics had negative impact on loss of psychological disorder, possibly leading to high prevalence of depression in this study. Additionally, the difference could result from using the different the assessment tool chosen for screening depression. Depression in this study is assessed by Zung SDS. This self-rating scale may assign symptoms experienced in dialysis populations (such as fatigue, poor appetite, and sleep disturbance) as the symptoms of depression and may overestimate depression prevalence.

In this study, decreased hemoglobin level was risk factor for depressive symptoms in CAPD patients, which are consistent with previous findings in healthy adults [18–20]. One meta-analysis study included 32 792 378 women showed that anemia was a significant risk factor for maternal depression [18]. Vulser et al. reported a study examining the association between anemia and depression in adults free of chronic disease and medication from the general population including 44 173 healthy participants [19]. They found depressed participants had significantly higher risk of anemia compared to non-depressed participants, after adjustment for sociodemographic and health-related variables. In international samples of older adults, anemia, as well as the severity of anemia, were independent risk factors for depression [20]. The present study also found that serum hemoglobin level was independently associated with depressive disorder in peritoneal dialysis patients. Improving anemia is the essential measure to ameliorate the psychological disorder of CAPD patients.

Several plausible mechanisms have been posited to explain the relation between anemia and depression. Anemia may lead to depressive symptoms. First, lower hemoglobin reduces blood oxygen carrying capacity [21], which may induce cerebral hypoxia and thereby contribute to anemic cerebral dysfunction. It was reported that anemia induced cerebral atrophy and might lead to permanent neurological injury [22]. Additionally, anemia is associated with compensatively higher regional cerebral blood flow in frontal, middle temporal, and hippocampal regions which are entities in the depression pathways [23]. Furthermore, anemia may induce dyspnea and fatigue, which in turn reduce social activity and may contribute to the onset of depressive symptoms [24, 25]. Conversely, low hemoglobin levels may occur as a consequence of depression. Depressed patients usually intake unhealthy dietary and may lead to iron, vitamin B12 and folic acid deficiencies that contribute to anemia [26, 27].

The present study has some limitations. First, the validity of SDS has been established in clinical depression evaluation [28, 29]. However, the self-report scales may overestimate depression presence, particularly in the dialysis patients [8]. Second, other factors that could have been associated with depression, such as the inflammatory markers, sleep disorders, social support, economic status were not included in this analysis. Third, the patients were recruited from a single center, and the small sample size limited the generalizability of the findings. The present study is a regional study, and it could be that this work may not generalize to other regions of China, nor to other countries with certain (unspecified) characteristics that distinguish the setting from this study’s. Finally, owing to the cross-sectional design of this study, no causal directions in the association between depression and anemia could be drawn. Longitudinal studies are warranted to assess the relationship between anemia and depression.

## Conclusion

This study has shown the occurrence of depression was independently associated with serum hemoglobin in CAPD patients even after adjustment for a wide range of sociodemographic and clinical characteristics. Although no conclusion could be drawn about causality, these findings suggest that CAPD patients with anemia should be screened for depression. It highlights the need to improve multiple aspects of end-stage renal disease management, including early diagnosis and treatment of anemia.

## Data Availability

The information and data of the study population were extracted from Hospital Information System. The datasets are not publicly available because the individual privacy of the participants should be protected. Data are however available from the corresponding author on reasonable request.
